# Detection of Differentially Methylated Regions Using Bayes Factor for Ordinal Group Responses

**DOI:** 10.3390/genes10090721

**Published:** 2019-09-17

**Authors:** Fengjiao Dunbar, Hongyan Xu, Duchwan Ryu, Santu Ghosh, Huidong Shi, Varghese George

**Affiliations:** 1 Genomics Research Center, AbbVie, North Chicago, IL 60064, USA; fengjiao.dunbar@abbvie.com; 2 Department of Population Health Sciences, Augusta University, Augusta, GA 30912, USA; hxu@augusta.edu (H.X.); sghosh@augusta.edu (S.G.); 3 Department of Statistics and Actuarial Science, Northern Illinois University, DeKalb, IL 60178, USA; dryu@niu.edu; 4 Georgia Cancer Center, Augusta University, Augusta, GA 30912, USA; hshi@augusta.edu

**Keywords:** Bayes factor, Bayesian mixed-effect model, CpG sites, DNA methylation, Ordinal responses

## Abstract

Researchers in genomics are increasingly interested in epigenetic factors such as DNA methylation, because they play an important role in regulating gene expression without changes in the DNA sequence. There have been significant advances in developing statistical methods to detect differentially methylated regions (DMRs) associated with binary disease status. Most of these methods are being developed for detecting differential methylation rates between cases and controls. We consider multiple severity levels of disease, and develop a Bayesian statistical method to detect the region with increasing (or decreasing) methylation rates as the disease severity increases. Patients are classified into more than two groups, based on the disease severity (e.g., stages of cancer), and DMRs are detected by using moving windows along the genome. Within each window, the Bayes factor is calculated to test the hypothesis of monotonic increase in methylation rates corresponding to severity of the disease versus no difference. A mixed-effect model is used to incorporate the correlation of methylation rates of nearby CpG sites in the region. Results from extensive simulation indicate that our proposed method is statistically valid and reasonably powerful. We demonstrate our approach on a bisulfite sequencing dataset from a chronic lymphocytic leukemia (CLL) study.

## 1. Introduction

It is now widely accepted that cancer develops through a series of stages [1]. It starts from a very limited area, not invasive and metastatic at the early stage, then spreads to distant sites in the body, and becomes highly invasive and metastatic at the late stage. In addition, patient survival times are significantly reduced at the late stages. For example, the 5-year relative survival rate for lung cancer is 54% at a localized stage, and is reduced to 4% at the distant stage [2]. More than half of lung cancers are diagnosed at a distant stage, which indicates that early diagnosis of cancer is the main factor to enhance patient survival. Therefore, markers for early detection and proper classification of the tumor are extremely critical to improve life expectancy. Furthermore, identifying high-risk cancer patients at an early stage, would allow them to receive standard chemotherapy in advance.

DNA methylation has been found to be a marker for disease diagnosis, such as in cancer [3]. Significant progress has been made using DNA methylation differences to capture substantial information about the molecular and gene-regulatory states among biology subtypes, such as tumor and normal tissues [4].

In addition, DNA methylation can be used as a marker to differentiate disease severity, such as early and late stages in breast cancer [5], ovarian cancer [6] and prostate cancer [7]. Most of them have potential functions in inducing and suppressing cancer metastasis. Moreover, DNA methylation is associated with tumor size in colorectal cancer [8].Patients with higher methylation showed more frequent recurrence as compared with the low-methylation group, and shortened cancer-related survival and recurrence-free survival [8]. 

These findings show the critical importance of a better understanding of cancer progression and metastasis, which could help make better prediction of the clinical aggressiveness of cancer. Since DNA methylation is associated with disease severity, detecting differentially methylated regions (DMRs) can help understand cancer progression. 

Most analyses are conducted by creating dichotomies based on biological subtypes, such as early and late cancer stages, and then detect DMRs by comparing the differences of DNA methylation rates between two groups [5,6,7]. However, when there are actually more than two groups, such approaches may lose information regarding multiple disease status, due to collapsing or ignoring clinically relevant subtypes, resulting in suboptimal clinical conclusions and decisions. 

To use multiple disease status, it is possible to run multiple testing for the association between DNA methylation and multiple group responses, using the methods for two groups. Although we can simply run analysis for all pair-wise comparisons and combine the results, it is not trivial when considering the regional correlation of DMRs, and would increase the multiple testing burden. 

Another possible method is the generalized linear model that includes indicator variables for different levels of disease status. This method has the advantage that it can adjust for covariates. However analysts are often faced with noisy estimates of category-specific regression coefficients, which can lead to unreasonable patterns in the regression coefficients corresponding to different levels of disease status, and it can reduce the power [9]. 

To improve the efficacy of an overall test, one can take advantage of the fact that cancer develops through a series of stages, or different levels of disease severity in general, and develop statistical methods that can incorporate the ordering of disease status. However, the widely used trend test is not an ideal method, because it requires scores or weights for different levels of disease status, which are generally unknown.

Here we propose a Bayesian approach and use the Bayes factor to test the association between methylation rates and disease severity. The proposed Bayes Factor Method (BFM) can incorporate monotonicity constraints, and find DMRs in which methylation rates increase (or decrease) as the diseases become more severe. Patients are classified into groups based on the disease severity (e.g., stages of cancer), and DMRs are detected by using moving windows along the genome. Within each window, the Bayes factor is calculated and is used to test the hypothesis of constant versus monotonic increase in methylation rates corresponding to the severity of the disease.

In addition, since DNA methylation rates have been shown to be correlated at nearby CpG sites with complicated correlation structure [10], a linear mixed-effect model is used to incorporate the correlation of methylation rates between and within CpG sites in the region. 

## 2. Materials and Methods 

### 2.1. Methods

Classical statistical inference under constrained parametric spaces has been addressed by many studies. Among them, Bartholomew [11] presented one of the first tests for K multinomial proportions with inequality constraints. He proposed a test of $H_{0}:p_{1}=p_{2}=\ldots =p_{K}$ against the simple ordered $H_{1}:p_{1}\leq p_{2}\leq \ldots \leq p_{K}$ with at least one strict inequality, where $p_{k}$ (k=1,2,…,K) represents the proportion the k^th^ group. Under $H_{0}$, the maximum likelihood estimator of $p_{k}$ is the overall sample proportion $\pi_{k}$. If the sample multinomial proportions satisfy $\pi_{1}\leq \pi_{2}\leq \ldots \leq \pi_{K}$, then the order-restricted ML estimator is ${\hat{p}}_{k}=\pi_{k}$. However, sometimes the sample proportions may not satisfy the ordering $\pi_{1}\leq \pi_{2}\leq \ldots \leq \pi_{K}$; in that case, calculation of the restricted maximum likelihood estimator (RMLE) is subject to arbitrary orderings of the parameters, and it requires specialized algorithms that are not easily generalizable [9].

Robertson and Wegman [12] proposed a likelihood ratio statistic for the inequality-constrained binomial problem, which compares parameters for independent samples from a single-parameter exponential family distribution. Before calculating the test statistic, they used the pool-adjacent-violators algorithm [13] to pool “out-of-order” categories for which $\pi_{k}>\pi_{k+1}$ until the resulting sample proportions are monotone increasing. The order-restricted ML estimators ${\hat{p}}_{k}$ become the adjusted sample proportions. 

The idea of applying an isotonic transformation to the unconstrained parameter estimates motivated Dunson and Neelon [9] to create a Bayesian alternative approach for this problem, which has been adapted here. They proposed to use Bayes factors for assessing ordered trends, which are calculated based on the output from Gibbs sampling. The samples from the order-constrained model are derived by transforming samples draws from an unconstrained posterior density using an isotonic regression transformation. Next, we explain our proposed Bayes factor method (BFM).

Suppose $m_{\mathrm{kij}}$ is the count of methylated molecules at CpG site j of individual i in group k. We assume $m_{\mathrm{kij}}\sim B\left(c_{\mathrm{kij}},p_{\mathrm{kij}}\right)$, where $c_{\mathrm{kij}}$ is the coverage, and $p_{\mathrm{kij}}$ is the true methylation rate at that particular site, with k=1,2,…, K, $i=1,2,\ldots ,{\;n}_{k}$ and j=1,2,…,m.

Within each moving window along the genome, a mixed-effect model is considered to allow the correlation of methylation rates between and within CpG sites. The logit link function for the methylation rate $p_{\mathrm{kij}}$ is expressed by

(1) $$\mathrm{logit}\left(p_{\mathrm{kij}}\right)=\mu_{k}+\nu_{0\mathrm{ki}}+\nu_{1\mathrm{kij}},$$

where $\nu_{0\mathrm{ki}}$ and $\nu_{1\mathrm{kij}}$ are the random effects. The random effect $\nu_{0\mathrm{ki}}\sim N\left(0,\sigma_{\nu_{0}}^{2}\right)$ is used to model the interindividual correlation of methylation rates within each CpG site, while the random effect $\nu_{1ki}={\left(\nu_{1\mathrm{ki}1},\nu_{1\mathrm{ki}2},\;\ldots ,{\;\nu }_{1\mathrm{kim}}\right)}^{T}\sim N\left(\mu_{0},\Sigma \right)$, with $\mu_{0}={\left(0,0\ldots 0\right)}^{T}$ is used to model the correlation of methylation rates between CpG sites. 

Here $\mu_{k}$ in (1) is the fixed effect for each group, representing the association between methylation rates and group responses. The strength and direction of the association is modeled by prior distribution $N\left(\mu_{\mu },\sigma_{\mu }^{2}\right)$, which means the parameters of $\mu_{\mu }$ and $\sigma_{\mu }^{2}$ control the distribution of $\mu_{k}$, and implies that all of the methylation rates are drawn from a common distribution. This brings the advantage of allowing for heterogeneity of effects across CpG sites, instead of just pooling information across CpG sites in a region. Pooling assumes that each CpG site in the region has same methylation rates, while BFM considers the methylation rates of each CpG sites to be a random quantity governed by a prior distribution. 

With assigned hyperpriors $\mu_{k}\sim N\left(0,1000^{2}\right)$, $\sigma_{k}^{2}\sim \mathrm{IG}\left(1,100\right)$, $\sigma_{\nu_{0}}^{2}\sim \mathrm{IG}\left(1,100\right)$ and $\Sigma^{-1}\sim \mathrm{Wish}\left(I_{m},m\right)$ for m CpG sites in the moving window. The posterior distribution of $\mu_{k}$ is based on the mixed-effect logistic model (1), and it is used to calculate the Bayes factor for comparing the two models, $M_{0}:\mu_{1}=\mu_{2}=\ldots =\mu_{K}$, $M_{1}:\mu_{1}\leq \mu_{2}\leq \ldots \leq \mu_{K}$ with at least one strict inequality, in order to see whether there is an ordered constraint of methylation rates corresponding to severity of the disease. 

To calculate the Bayes factor, first we drew samples $\mu_{1},\mu_{2},\ldots ,{\;\mu }_{K}$ from the posterior distribution by using Gibbs sampling. After that, an isotonic transformation is used to transform $\mu_{1},\mu_{2}\ldots {\;\mu }_{K}$ into ${\tilde{\mu }}_{1},{\tilde{\mu }}_{2},\ldots ,{\tilde{\mu }}_{K}$, with ${\tilde{\mu }}_{1}\leq {\tilde{\mu }}_{2}\leq \ldots \leq {\tilde{\mu }}_{K}$ [8] by using the min-max formula for the isotonic transformation, given by,

(2) $${\tilde{\mu }}_{k}=g_{k}\left(\mu \right)=\underset{t\in U_{k}}{\min}\underset{s\in L_{k}}{\max}\left(\frac{1_{t-s+1}^{\prime }V_{\left[s:t\right]}^{-1}\mu_{\left[s:t\right]}}{1_{t-s+1}^{\prime }V_{\left[s:t\right]}^{-1}1_{t-s+1}}\right)\;\mathrm{for}\;j=1,2,\ldots ,\;K,$$

where ***V***=diag*V*_1_,..., *V_K_* denotes the posterior covariance matrix and the diagonal submatrix V_i_, i = 1, …, k, is the covariance matrix of the i^th^ ordered group. It is estimated from the samples of the posterior density of μ. $U_{k}$ and $L_{k}$ denote subsets of {1,…,K} such that the ordering $\mu_{j^{\prime }}\leq \mu_{j}$ for all $j^{\prime }\in L_{k}$ and the ordering $\mu_{j^{\prime }}\geq \mu_{j}$ for all $j^{\prime }\in U_{k}$. Also samples $\mu_{1}^{0},\mu_{2}^{0},\ldots ,{\;\mu }_{K}^{0}$ are drawn from the prior density and transformed into ${\tilde{\mu }}_{1}^{0},{\tilde{\mu }}_{2}^{0},\ldots ,{\tilde{\mu }}_{K}^{0}$, with ${\tilde{\mu }}_{1}^{0}\leq {\tilde{\mu }}_{2}^{0}\leq \ldots \leq {\tilde{\mu }}_{K}^{0}$, by using the isotonic transformation in (2). The Bayes factor for each window (with moving windows along the genome) is given by,

$$\mathrm{BF}=\frac{P\left(M_{1}|\mathrm{data}\right)/P\left(M_{1}\right)}{P\left(M_{0}|\mathrm{data}\right)/P\left(M_{0}\right)}=\frac{P({\tilde{\mu }}_{K}>{\tilde{\mu }}_{1})/P({\tilde{\mu }}_{K}^{0}>{\tilde{\mu }}_{1}^{0})}{P\left({\tilde{\mu }}_{K}={\tilde{\mu }}_{1}\right)/P\left({\tilde{\mu }}_{K}^{0}={\tilde{\mu }}_{1}^{0}\right)}$$

Please note that the isotonic transformation in (2) changes our hypotheses slightly, making the resulting Bayes Factor an approximation rather than exact [14]. The windows with highest value of the Bayes factor among all windows are used for evaluating DMRs. 

Thus, the Bayes factor is the ratio of the marginal densities of the data under the two hypotheses, and it can be used to weigh evidence in favor of a hypothesis, by utilizing all the information contained in the full likelihood. Our proposed BFM can detect DMRs associated with disease severity, especially detecting DMRs with monotonically increasing or decreasing methylation rates, as the disease severity increase. It uses a mixed-effect model to not only adjust for correlation of methylation rates between CpG sites within each moving window but also correlations within CpG sites. 

In addition, by adding covariates $x_{\mathrm{ki}}$ in the model (1), we can account for the effects of covariates that are associated with methylation rates, such as age [15] and gender [16]. 

To aid in the interpretation of the Bayes factor, Jeffreys [17] proposed the following rule of thumb: “When 3 < BF ≤ 10 the evidence is positive, when 10 < BF ≤ 100 the evidence is strong, and when BF > 100, the evidence is decisive”. As Kass and Raftery [18] pointed out, these categories are not precise calibration, but rather a descriptive statement about the standards of evidence in scientific investigations. 

### 2.2. Simulation Study of the Properties of BFM

Extensive simulation was conducted to study the statistical validity and power of BFM to detect DMRs. For simplicity, for each individual, we simulated one CpG island (genomic region with CpG sites) consisting of m equally spaced CpG sites, with only one DMR of length r(<m) in the middle of the island. Further, we used equal sample size, N, for each of the K groups, and, we did not include any covariates. 

Simulation Setup:

The goal here is to simulate methylation rate at each CpG site for each individual. This is achieved in two steps. In step 1, methylation data in the form of NGS short reads sequences were simulated for each CpG site, with correlated methylation status between CpG sites. We also assumed that methylation status at CpG sites among different sequences were independent, as expected in NGS data. In step 2, the individual methylation rates were calculated by summarizing the methylation status at each CpG site from the short read sequences.

The simulation details are described below:

First, we generated 100 NGS short reads using 100 pairs of random numbers {a, c} where a is the start point and c is the length of each short read sequence. 

Then we used vector Y = $\left(Y_{\mathrm{kis},a},{\;Y}_{\mathrm{kis},a+1},\ldots ,{\;Y}_{\mathrm{kis},a+c-1}\right)$ to define the methylation status for short read sequence s of individual i in group k, and generated Y from a multivariate Bernoulli distribution to allow for the correlation among the methylation rates.$P\left(Y=y\right)=P\left(y_{\mathrm{kisa}},{\;y}_{\mathrm{kis},a+1},\ldots ,{\;y}_{\mathrm{kis},a+c-1}\right)$ of such a discrete random vector Y depends on $2^{c}$ probabilities, p(0,0,…,0), p(0,0,…,1), …, p(1,1,…,1), specific to the different realizations of Y. Considering the fact that if a vector $\left(Y_{1},{\;Y}_{2},\ldots ,{\;Y}_{p}\right)$ follows p-variate Bernoulli distribution, the conditional distribution of $\left(Y_{1},{\;Y}_{2},\ldots ,{\;Y}_{r}\right)$ (r<p) given $\left(Y_{r+1},{\;Y}_{r+2},\ldots ,{\;Y}_{p}\right)$ is also a multivariate Bernoulli distribution [18]. We can utilize this fact to reduce the dimensionality of the unconditional multivariate Bernoulli distribution.

Because of the correlation of methylation rates between CpG sites, we treated methylation status $Y_{\mathrm{kis},j}$ at each CpG site j on short read sequence s as a branching process, taking advantage of the property of multivariate Bernoulli distribution [19]. We assumed that, for CpG site j, branching probabilities were the same for each short read sequence of all individuals in group k. Thus, we defined the branching probability $p_{\mathrm{kj}}$ = $P\left(Y_{\mathrm{kis},j}=1{|Y}_{\mathrm{kis},j-1}=1\right)$ as the probability of methylated sequence read at CpG site j, conditional on the methylated sequence read at CpG site j−1 on the same short read sequence of the same individual. Similarly, we defined the branching probability $q_{\mathrm{kj}}$ = $P\left(Y_{\mathrm{kis},j}=1{|Y}_{\mathrm{kis},j-1}=0\right)$ as the same probability, conditional on unmethylated sequence read at CpG site j−1.

The methylation status $\left(Y_{\mathrm{kis},a},{\;Y}_{\mathrm{kis},a+1},\ldots ,{\;Y}_{\mathrm{kis},a+c-1}\right)$ were generated as follows:

For the first CpG site of the sequence, the methylation status $y_{\mathrm{kisa}}$ was generated from Bernoulli distribution $\mathrm{Bern}\left(m_{a}\right)$, with $m_{a}=\left(p_{\mathrm{ka}}+q_{\mathrm{ka}}\right)/2$.

The methylation status $y_{\mathrm{kis},j}$ for j=a+1, …, a+c−1 was generated with $y_{\mathrm{kis},j}\sim \mathrm{Bern}\left(p_{\mathrm{kj}}\right)$ if $y_{\mathrm{kis},j-1}=1$ or $y_{\mathrm{kis},j}\sim \mathrm{Bern}\left(q_{\mathrm{kj}}\right)$ if $y_{\mathrm{kis},j-1}=0$.

After generating all the sequences at every CpG site for each individual, we calculated the total numbers of methylated and unmethylated short read sequences at CpG site j for individual i in group k, $\underset{s}{\sum }\left(y_{\mathrm{kis},j}=1\right)$ and $\underset{s}{\sum }\left(y_{\mathrm{kis},j}=0\right),$. Then the methylation count and the sequencing coverage are given by $m_{\mathrm{kij}}=\underset{s}{\sum }\left(y_{\mathrm{kis},j}=1\right)$ and $c_{\mathrm{kij}}=\underset{s}{\sum }\left(y_{\mathrm{kis},j}=1\right)+\underset{s}{\sum }\left(y_{\mathrm{kis},j}=0\right),\;$ respectively.

We generated one CpG region with 24 CpG sites for each individual, 6 of which (from site 10 to 15) constituting the DMR. We simulated four groups of severity levels, with sample size 50 in each group, and repeated it with sample size 100. The branching probabilities, $p_{\mathrm{kj}}$, were pre-determined. Also, we chose $q_{\mathrm{kj}}=p_{\mathrm{kj}}-0.2$. We also simulated two different scenarios of DMR patterns. 

Under Scenario 1, we chose the probabilities, $p_{\mathrm{kj},}$ to be symmetric around the middle of the DMR (CpG sites 12 and 13). The predetermined probabilities $p_{\mathrm{kj}}$ and their symmetric pattern under Scenario 1 are presented in Table 1.

Under Scenario 2, we randomly chose the CpG sites with the peak values of $p_{\mathrm{kj}}$ within the simulated DMR (between sites 10 and 15), varying it for different individuals. Specifically, for each individual in each group, we first generated a random number r (between 10 and 15) for the location of the CpG site with the highest methylation, and then chose the branching probabilities $p_{\mathrm{kj}}$ to increase from 10 to r and then decrease from r to 15. The $p_{\mathrm{kj}}$ for the non-DMCs remained the same as in Scenario 1. The second scenario is a more realistic depiction of the real world. However, the results and conclusions should be the same under both situations.

## 3. Results

### 3.1. Simulation Results

**Figure 1:**
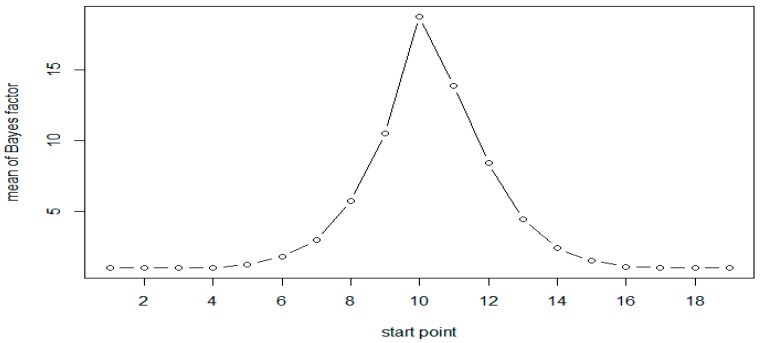
Mean of Bayes factors at each CpG site with *N* = 50 (Scenario 1).

**Figure 2:**
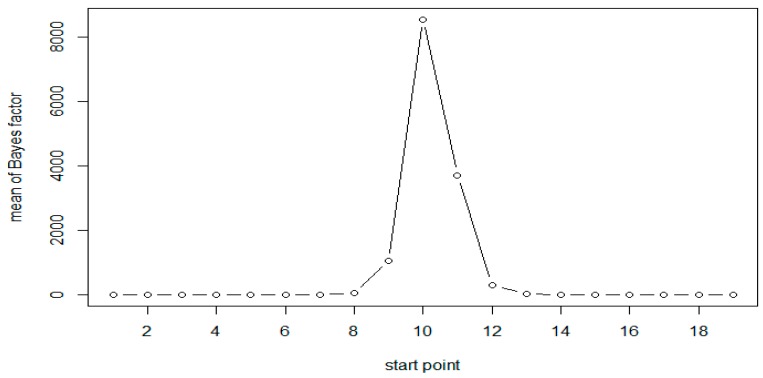
Mean of Bayes factors at each CpG site with *N* = 100 (Scenario 1).

A total of 1000 replicates were simulated. For each replicate, the Bayes factor was calculated for each moving window with window size of 6. Calculations were based on 3000 Gibbs samplers, with 1000 Gibbs samplers for the burn-in period. The results of simulation for both the scenarios are presented in Table 2. As expected, the results are very similar for both scenarios. The results of Scenario 1 are plotted in Figure 1 and Figure 2. As evident from Table 2, following Jeffreys’ rule, when the moving windows contain at least three of the six CpG sites, we have strong evidence of differential methylation when sample size of 50 in each group and decisive evidence when sample size is 100.

All results show that the Bayes factors reach their maximum in the simulated DMR (CpG sites 10–15). However, the Bayes factors are not symmetric, the windows on the right side of the peak have larger values compared to those on the left side. This is attributed to the fact that the methylation status at a given site was generated conditional on that at the previous site of the same sequence. As expected, when the sample size is doubled the Bayes factors and the evidence in support of methylation increases significantly, as seen in Table 2 and Figure 1 and Figure 2.

In order to illustrate that our proposed method is statistically valid and to ensure that the BF in our method is a meaningful measure for comparison with frequentist approaches, we computed Bayes factors exclusively for all moving windows that do not include the differentially methylated sites 10–11. Among these Bayes factors, 95% were less than 1.34 and 99% were less than 1.50, both consistent with Jeffreys’ rule. These values can be thought of as the cut-offs corresponding to 5% and 1% empirical type I error rates. We calculated the proportions of times the Bayes factors fall above these cut-offs, for all possible numbers of DMCs in the moving window. These results are given in Table 3. For the simulated data they are comparable to the conclusions based on frequentist interpretations of type I error and power. For the real data analysis, one could employ a permutation test to derive the cutoff values under the null hypothesis. However, since the frequentist interpretation is not necessarily consistent with the Bayesian conclusions, using Jeffrey’s rule for decision making may be more desirable when analyzing real data.

### 3.2. Data analysis

We used our proposed BFM to analyze methylation data from a genome-wide association study of chronic lymphocytic leukemia (CLL), which manifests as a result of clonal expansion of malignant B cells. B-cell lymphoma, mostly prevalent among adults, is a heterogeneous disease [20,21]. It is clinically important to find heterogeneity of patients at the molecular level, which can help design specific interventions for patients at different severity levels. 

Over the last decade, research in CLL has resulted in significant advances such as identification of several molecular alternations with prognostic values. These include specific cytogenetic patterns [22], mutational status of the immunoglobulin heavy chain variable gene (IgVH) [23] and expression of CD38 [24]. It has been found that patients lacking the mutation have a poorer prognosis. Patients with lower levels of CD38 have slower disease progression [23,25].

Several research groups have demonstrated that DNA methylation of multiple promoter-associated CpG islands is common in CLL [15,26,27]. Detection of aberrant DNA methylation in CLL could result in the development of an epigenetic classification of the disease with prognostic and therapeutic potential. 

CD19+ B cells from peripheral blood were collected from CLL samples and normal control subjects. All CLL samples were obtained from patients at the Ellis Fischel Cancer Center (EFCC), the Georgia Cancer Center of Augusta University and the North Shore-LIJ Health System in compliance with the local Institutional Review Boards [28]. 

Illumina sequencing reads were generated for each sample by using RRBS [29]. In total, 20–30 million reads were sequenced for each sample, and 63%–75% were successfully mapped to either strand of the human genome (hg18) [28]. The average sequencing depth per CpG was between 32x and 43x. Eventually RRBS provided counts of DNA molecules that were methylated or unmethylated at each CpG site, and overall methylation status of approximately 1.8–2.3 million CpG sites were determined consistently for each sample in the study [28].

Tong et al. [30] pointed out that aberrant DNA methylation associated with CLL were located more frequently on chromosome 19. Hence, we analyzed genome-wide methylation data on 17,917 CpG sites on Chromosome 19 of 40 patients.

### 3.3. Comparison of Bayesian Method with Scan Statistic Method for Two Groups

First, we tested for differential methylation under binary response, by dividing the samples into two groups based on CD38 level of 20 as the cut-off. We had 23 subjects with CD38 ≤ 20 and 17 subjects with CD38 > 20. BFM and Scan statistic method (SSM) [31] were compared, using moving windows with 10 CpG sites in each window. 

For comparing the two methods, we used a cut-off value of 2 for BFM and a 5% significance level for SSM. A total of 181 genes in DMRs were detected by SSM, and 183 genes were detected by BFM, using these criteria. Among these, 41 from SSM and 42 from BFM were found in PubMed publications as associated with leukemia (Table 4). There were 67 overlapping genes of which 18 were found in PubMed. They are ACP5, ATF5, BIRC8, C3, CARD8, CEACAM8, CERS1, CKM, CRTC1, IL4l1, LAIR1, MAP1S, NFIX, PDE4C, PLEKHG2, PLVAP, RFX1, and ZNF331 [32,33,34,35,36,37,38,39,40,41,42,43,44,45,46,47,48,49].

C3 and LAIR1((INK4a))genes were both detected, which were shown to be related to acute myeloid leukemia [34,41]. Actually, both C3 and LAIR1 genes connect with the transcription factor CREB (cyclic AMP response element binding protein), which has a role in the pathogenesis of AML and other cancers [50,51].

### 3.4. Bayesian Method for Ordinal Group Responses

To test whether the methylation rates increase as the CD38 levels increase, the samples were classified into four risk groups based on CD38 level, with 5 non-leukemia subjects in group 1, 23 patients in group 2 with CD38 ≤ 20, 9 patients in group 3 with 20 < CD38 ≤ 50, and 8 patients in group 4 with CD38 > 50. Though there are advantages of modeling CD38 as a continuous variable, but on the other hand, modeling as an ordinal variable is more robust to distributional assumptions. Again, moving windows of size of 10 were used for analysis. In fact, in clinical studies it is a common practice to put patients into discrete disease risk groups based on continuous measures.

Because of multiple testing issues associated with the comparison of four groups, we used a more stringent criterion of BF > 19 to evaluate the strength of evidence of differential methylation [8]. A total of 789 windows showed strong evidence of differential methylation using this criterion. The start and end positions in base pairs for each detected DMR were used in the UCSC genome browser to find the genes in the regions, and eventually 125 genes were found in these regions. Among them, 35 were associated with leukemia on PubMed literature. Some of these were not detected when only two groups were considered even with a less stringent criterion. They are BRD4, ELL, ERCC1, ERCC2, GDF15, JUND, POLD1, PRDX2, RANBP3, SPIB and TSPAN16 [52,53,54,55,56,57,58,59,60,61,62].

## 4. Discussion

Results from our simulation study indicate that BFM is a valid approach to detect DMRs when considering ordinal group responses, since the calculated Bayes factors were very large for simulated DMRs, and close to 1 for non-DMRs. The real data analysis based on the CLL data also demonstrated that BFM is a valid method that is able to detect DMRs with methylation rates increasing (or decreasing) as disease severity increases.

In addition to being able to account for ordering of group responses, BFM also has an advantage of allowing for heterogeneity of methylation effects across CpG sites by modeling the methylation rates with a prior. Methods such as the SSM pools information across variants in a region, assuming that each CpG sites in the region have the same methylation rates. 

BFM with mixed-effect regression, not only can allow for covariates, but also the correlation between CpG sites. It takes advantage of the flexibility of the Bayesian framework, including the use of prior information when available as well as computational convenience, and uses distributions such as the multivariate normal to incorporate the correlation structure with inverse Wishart distribution as the prior for the correlation matrix. 

One disadvantage of the BFM is that it assumes that methylation rates of CpG sites within each moving window are independent of those outside of the window, while the SSM accounts for the correlation along the whole genome. 

BFM used a moving window to help decide the location and length of DMRs. But practically, it is very difficult to know the exact length of DMRs. This limitation is very common in statistical genetics, not only for detecting DMRs, but also for detecting rare variants [63]. Cross validation or bootstrap approaches might help determine the window sizes. It could be possible to develop other methods, for example, using genes and promoters instead of moving windows, along with the BFM to detect DMRs. 

As described by George and Laud [64], the Bayes factor used in the context of testing hypotheses is a meaningful measure of evidence because it is a reasonably approximate factor by which the odds are increased by the data. With default priors that are essentially flat over a wide range of the relevant parameter space, the approach is similar to the likelihood-based inference. However, direct comparison between methods such as BFM based on the Bayes factor with frequentist approaches should be done with caution, as the Bayes factor classification for decision process is not a precise calibration, but rather a descriptive statement about the standards of evidence. Our proposed method is rather exploratory in nature, leading to a ranked list of sites for follow up for formal confirmation.

We developed the BFM, focusing only on DNA methylation data. However, large-scale cancer genomics projects such as TCGA (The Cancer Genome Atlas Research Network) are currently generating multiple layers of genomics data for early tumor, including DNA copy number, methylation, and mRNA expression. Similar statistical methods for integrated analysis and systematic modeling of these genomics data deserve further attention. 

## Figures and Tables

**Table 1:** Conditional probabilities p_kj_ at each CpG site for simulation of BFM under Scenario 1.

Site	1	2	…	9	10	11	12	13	14	15	16	17	…	24
group 1	0.44	0.46	…	0.6	0.62	0.64	0.66	0.66	0.64	0.62	0.6	0.58	…	0.44
group 2	0.44	0.46	…	0.6	0.72	0.74	0.76	0.76	0.74	0.72	0.6	0.58	…	0.44
group 3	0.44	0.46	…	0.6	0.82	0.84	0.86	0.86	0.84	0.82	0.6	0.58	…	0.44
group 4	0.44	0.46	…	0.6	0.92	0.94	0.96	0.96	0.94	0.92	0.6	0.58	…	0.44

**Table 2:** Mean Bayes factors at each CpG site, based on simulation studies.

Start	End	N = 50 (Scenario 1)	N = 100 (Scenario 1)	N = 50 (Scenario 2)
1	6	1.02	1.02	1.03
2	7	1.01	1.02	1.01
3	8	1.01	1.02	1.02
4	9	1.02	1.01	1.01
5	10	1.24	1.53	1.26
6	11	1.78	3.12	1.78
7	12	2.95	9.16	2.85
8	13	5.74	41.42	4.95
9	14	10.53	1052.07	9.31
10	15	18.79	8554.12	18.31
11	16	13.9	3718.77	13.79
12	17	8.44	306.07	8.12
13	18	4.43	21.91	4.5
14	19	2.4	5.66	2.6
15	20	1.52	2.22	1.6
16	21	1.07	1.11	1.07
17	22	1.03	1.04	1.02
18	23	1.01	1.03	1.02
19	24	1.03	1.03	1.01

**Table 3:** Proportions of Bayes factors that fell above the cut-off.

Cut-off Point	Number of DMCs in the Windows						
	0	1	2	3	4	5	6
1.34	0.050	0.56	0.97	1	1	1	1
1.5	0.010	0.35	0.91	1	1	1	1

**Table 4:** Comparison of BFM and SSM for window size of 10 (*p* < 0.05).

	BFM > 2	SSM (*p* < 0.05)	Common
Total	183	181	67
PubMed	42	41	18
